# Decreasing the use of edible oils in China using WeChat and theories of behavior change: study protocol for a randomized controlled trial

**DOI:** 10.1186/s13063-018-3015-7

**Published:** 2018-11-16

**Authors:** Rui Zhu, Xianglong Xu, Yong Zhao, Manoj Sharma, Zumin Shi

**Affiliations:** 1 School of Public Health and Management, Chongqing Medical University, Yixueyuan Road, Yuzhong District Chongqing, Chongqing, CN 400016 China; 2 Research Center for Medicine and Social Development, Chongqing Medical University, Chongqing, 400016 China; 3 The Innovation Center for Social Risk Governance in Health, Chongqing Medical University, Chongqing, 400016 China; 4 Department of Behavioral and Environmental Health, School of Public Health, Jackson State University, 350 West Woodrow Wilson Avenue, Jackson, MS 39213 USA; 5 Health for All, Omaha, NE 68144 USA; 6 College of Health Sciences, Walden University, Minneapolis, MN 55401 USA; 7 Human Nutrition Department, Qatar University, Room E211, Building C01, Doha, PO Box 2713 Qatar

## Abstract

**Background:**

The consumption of edible oils in China has increased rapidly in recent years, and the total amount of edible-oil intake in the country has ranked first in the world. The choice and intake of edible oils, as a source of fats, are important factors that affect people’s health. Many chronic diseases are closely associated with high-calorie and saturated-fat intake. The influence of traditional concepts that promote the use of edible oils among women, particularly housewives, plays a key role in a household’s diet and nutrition because the diet-related knowledge, attitude and behaviour of housewives are dominant factors in planning and preparing their family’s meals. WeChat, which was developed by Tencent, is a multipurpose messaging, social media and mobile payment application (app) in China. Described by Forbes as one of the world’s most powerful apps, WeChat provides considerable convenience in disseminating knowledge. Accordingly, this study aims to design a pilot intervention to decrease the use of edible oils in Chinese households. The intervention, which is based on theories of behaviour change, will be implemented through WeChat.

**Design and methods:**

The study design is a randomised controlled trial that adopts knowledge, attitude and practice, social cognitive and stages of change theories as theoretical models. A total of 800 housewives between the ages of 25 and 45 years will be recruited on WeChat and from the communities in four areas (including rural and urban) in Chongqing, China. A self-administered questionnaire will be used to collect information regarding age, educational level, occupation, family members, edible-oil intake habits, knowledge of edible oils and WeChat usage habits. A total of 200 participants will be selected and randomly assigned to two equal-sized groups: group A (the intervention group) and group B (the control group). Group A will receive health education regarding edible oils for four consecutive weeks, whereas group B will be treated as the blank control. Each participant will complete a battery of knowledge, attitude and behaviour tests immediately, 3 months and 6 months after the intervention. In addition, weight, moisture rate, fat rate, visceral fat level and body mass index will be calculated using a multifunctional weighing scale, namely, Tanita BC-601 (Japan). The study is currently in the design stage.

**Discussion:**

This study aims to increase knowledge and awareness of the appropriate use of edible oils, thereby encouraging participants to change behaviour by decreasing the intake of unhealthy levels of edible oils. It will be the first intervention to investigate the use of edible oils in China through WeChat. We predict that receiving health education regarding edible oils through WeChat will substantially improve the knowledge and attitude of the respondents. The members of the intervention group will have increased awareness and will be willing to decrease their use of edible oils to remain healthy. Results of this study may provide scientific evidence for the effect of health education through WeChat on edible oil-intake behaviour, thereby offering a comprehensive intervention to decrease the use of edible oils and promote a healthy lifestyle.

**Trial registration:**

Chinese Clinical Trial Registry (primary registry in the World Health Organisation registry network): ChiCTR-IOR-17013472. Registered on 21 November 2017.

**Electronic supplementary material:**

The online version of this article (10.1186/s13063-018-3015-7) contains supplementary material, which is available to authorized users.

## Background

Edible oils are important sources of energy and essential fatty acids for the human body. An appropriate amount of edible-oil intake can provide humans with various types of fatty acids and accelerate the absorption of fat-soluble vitamins [1]. Nutritional, biological, clinical and medical studies postulate that fatty acids in edible oils play an important role in maintaining human health [2, 3]. For example, omega-3 fatty acids, which are polyunsaturated fatty acids, can promote the reduction of triglycerides and be beneficial for cardiac health. Studies have found that an inadequate intake of omega-3 fatty acids is a risk factor for many nutrition-related diseases [4]. Furthermore, the excessive consumption of edible oils will lead to too much calorie intake, which may be linked to various negative health consequences, such as obesity, diabetes and other chronic diseases, according to a previous study [5].

Edible-oil intake and dietary habits play crucial roles in human health [1]. At present, however, local and overseas studies on edible oils provide conflicting results. The Centres for Disease Control and Prevention reported that over one third of adults in the United States (approximately 78 million people) are suffering from obesity. The 2015–2020 Dietary Guidelines for Americans recommend that consuming low-fat dairy helps control fat intake [6, 7]. The obesity rate in Japan is extremely low compared with that in Europe and the United States, one of the important reasons is that Japanese people have less oil intake [8]. The World Health Organisation recommends that total fat intake should not exceed 30% of the total energy intake to avoid unhealthy weight gain [9]. Evidence indicates that oil intake in the Chinese diet is significantly beyond the standard. The per capita oil intake among Chinese reaches 42 g, which is considerably higher than the recommended 25 g; such excessive intake has become a major health obstacle [6, 10]. At present, the major types of edible oils in China are soybean oil, rapeseed oil and peanut oil, supplemented by animal oil; differences in oil intake have been observed among various populations and regions [11, 12]. For example, edible-oil intake is higher among men than among women [13]. Rural residents mostly use rapeseed oil, whereas rapeseed oil consumption is relatively low in urban areas [14]. Furthermore, Chinese people tend to prefer refined edible oils, which lack trace elements due to excessive refining, and thus, negatively affect immunity and health [1, 15].

Housewives are key figures in the current study. In China, women pay more attention to their family’s diet and nutrition than men, and as influenced by traditional ideas, housework is primarily the women’s duty [16]. At present, full-time housewives are not as common as before, particularly in larger cities and other urban areas [17]. Modern women seek employment not only to earn money to support their family but also for self-improvement. Accordingly, the housewives referred to in this study are women who play a key role in housework, particularly in cooking, in addition to their jobs. Besides, the knowledge, attitude and behaviour of women regarding nutrition also affect their children’s growth and health [18] because women play a dominant role in determining the diet and nutrition of their family [19]. However, no study has yet investigated the knowledge and attitude of Chinese housewives regarding edible oils or has intervened on their edible-oil intake behaviour. Therefore, the knowledge and attitude of housewives regarding excessive edible-oil intake should be assessed to promote healthy behaviour in choosing edible oils.

In recent years, China has witnessed an unprecedented increase in the use of social media. WeChat, which was released in January 2011, is a mobile communication tool developed by Tencent in China. WeChat is one of the leading social networks worldwide, ranking fifth in terms of the number of active users. As of 2018, it is one of the world’s largest stand-alone mobile apps, with over one billion monthly active users (902 million daily active users) [20]. WeChat has been dubbed China’s ‘app for everything’ and a ‘super app’ because of its wide range of functions and platforms. For comparison, Facebook Messenger and WhatsApp Messenger (two competitive international messaging services that are popular in the West) have approximately one billion monthly active users in 2017 but did not offer most of the other services available on WeChat [21]. WeChat exhibits a mass media feature, which can quickly send voice messages, videos, pictures and texts and allows repeatable views. Consequently, effective education can be provided at any time through WeChat, thereby saving considerable material resources. In addition, the Internet provides timely information and new features to attract respondents. Considering the aforementioned advantages of WeChat, we hypothesise that respondents will be more willing to participate in the project if WeChat is used than if traditional methods for delivering intervention are used. Hence, we speculate that this online method may reduce the dropout rate of respondents.

At present, public awareness of the risks of excessive oil consumption is weak, and most housewives lack the necessary knowledge regarding appropriate amounts of edible-oil intake [22]. Although numerous health information sources are readily available, distinguishing the authenticity of information is generally difficult given that some sources provide erroneous information. Most studies [23–25] have analysed the ingredients of edible oils and have investigated their pathogenic mechanism in various diseases. However, surveys on public cognition and factors associated with edible oils remain limited. Thus, decreasing the use of edible oils should be explored, particularly under the current condition of increased consumption. Moreover, a scientific dissemination platform on WeChat may be used as a nutritional education carrier, which is urgently needed. Furthermore, considering its enormous number of users, WeChat may become an effective intervention tool for decreasing the use of edible oils among households.

## Methods and analysis

### Study design

**Fig. 1:**
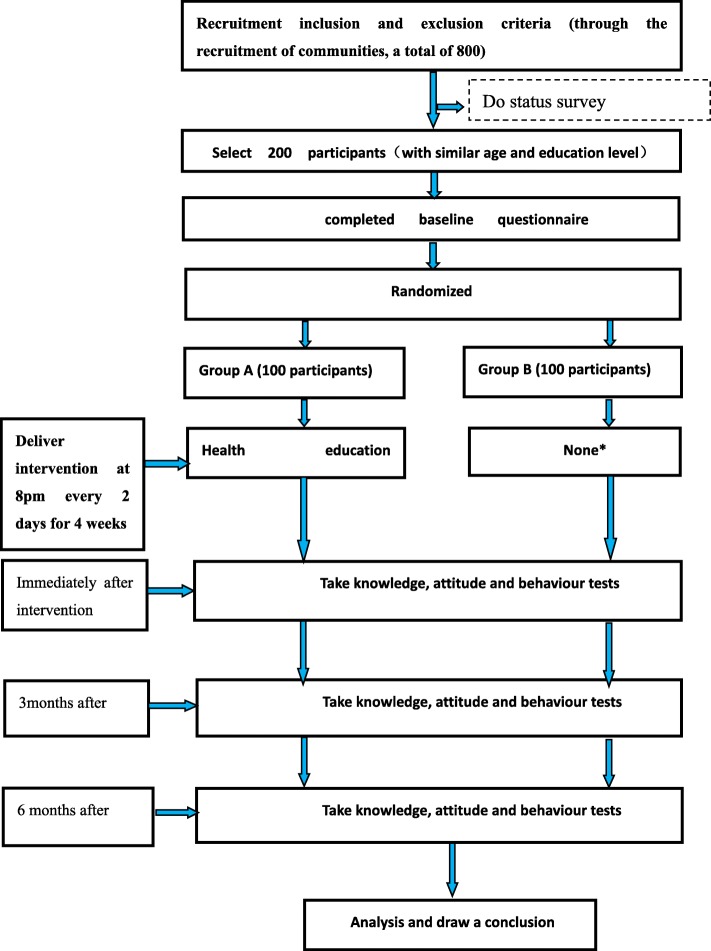
Flowchart of the study design. *After 6 months of follow-up, the control group will be provided with the same information that was given to the intervention group to raise the awareness of reducing their edible oil intake

**Fig. 2:**
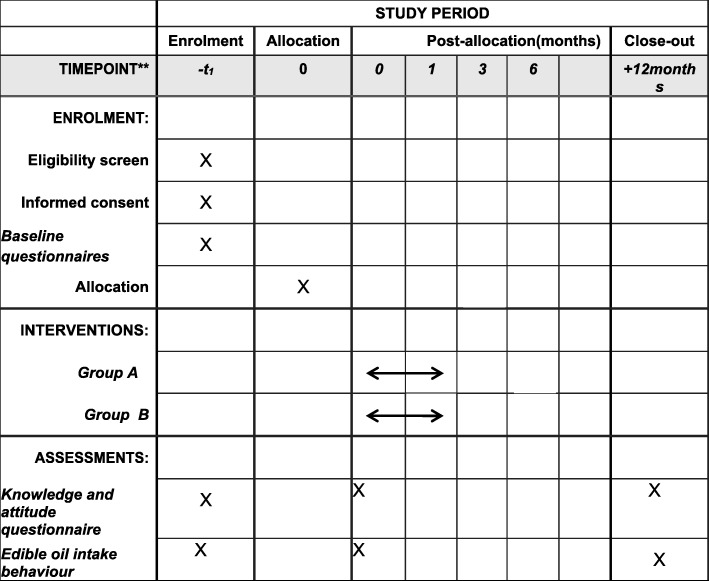
Standard Protocol Items: Recommendations for Interventional Trials (SPIRIT) Schedule of enrolment, assessment and interventions

This study has three phases. First, 800 participants will be recruited from different communities to answer the status survey questionnaire, which allows us to obtain information regarding the current consumption of edible oils and the use of WeChat. Subsequently, 200 participants will be selected from the status survey. After the baseline survey, participants who met the inclusion criteria and provided demographic information will be randomly divided following a 1:1 ratio to an intervention group and a control group using computerised simple randomisation in SPSS. An intervention will then be performed on the intervention group for 4 weeks. Afterwards, tests will be conducted on each group immediately, 3 months and 6 months after the intervention. Furthermore, after 6 months of follow-up, the control group will be provided with the same information given to the intervention group to raise the awareness of its members regarding the need to reduce their edible-oil intake. This study has been registered at the Chinese Clinical Trials Registry (ChiCTR-IOR-17013472). Figure 1 shows the research design. Figure 2 shows the schedule of enrolment, assessment and intervention. Additional file 1 provides the complete Standard Protocol Items: Recommendations for Standard Protocol Items: Recommendations for Interventional Trials (SPIRIT) Checklist.

### Study hypothesis

The knowledge of participants who receive health education regarding edible oils through WeChat will increase and they will be more willing to decrease their use of edible oils and change their unhealthy cooking and eating behaviour compared with those who have not received the intervention.

### Sample size calculation and participants

Previous studies have indicated that the daily per-capita excessive edible-oil intake (the standard value is 25–30 g/day) in households is 79.6% [26]. We set *p* = 0.8, *q* = 1 − *p* = 1–0.8 = 0.2, margin of error *d* = 0.1, *p* = 0.08, *α* = 0.05 and *Z*_*α*2_= 1.96. The sampling size is *N*= $\frac{{Z_{\alpha /2}}^2\times pq}{d^2}$ =1.96^2^ × 0.8 × 0.2/0.08^2^ = 96.04 families, and considering that the sample population in this item is female, then *N* ≈ 200. The participants’ non-response rate is 10%, and the questionnaire’s qualified rate is 90%; thus, the total required sample size is *N* ≈ 250. Finally, to obtain more comprehensive cross-sectional data, we expand the sample size by three times and recruit *N* = 3 × 250 ≈ 800 participants for the status survey.

During the intervention stage, we will select 200 participants to participate in a baseline survey from the status survey, which is based on their age and education to ensure that the demographic characteristics will have no significant difference. Subsequently, they will be divided randomly into an intervention group (group A) and a control group (group B) to conduct the intervention.

### Participants and recruitment

Participants will be recruited from the following four areas (rural and urban) in Chongqing, China: Yuzhong District, Shapingba District, Dianjiang District and Wulong District. Each district represents a different level of economic development.

A publicity group will be set up during the early stage of the project. An advertisement with a short comic strip will be designed to present the study, and printed posters and leaflets will be disseminated in the cooperating communities. Women who are interested can register at the community centre. Community leaders can, if they find it appropriate, inform them about the possibility of participating in the study. Recruitment will also be done through announcements on microblogs and WeChat. Women who wish to participate will have to provide their informed consent and enrol on the study’s homepage if they meet the inclusion criteria.

The inclusion criteria are as follows: women who are between the ages of 25 and 45 years, involved mainly in housework (particularly cooking) and active daily users of WeChat (i.e. using WeChat more than three times daily).

The exclusion criteria are as follows: individuals who have never cooked, have been involved in other edible-oil experiments and those with cognitive or verbal impairments or diseases that require them to follow strict diets.

### Theoretical models

We adopt the knowledge, attitude and practice (KAP), social cognitive and stages of change (SOC) theories as theoretical models. These models are frequently used to help change health-related behaviours, such as tobacco cessation, prevention of chronic diseases and nutritional education [27–29]. A health education intervention regarding edible oils will be conducted through the WeChat platform, such that housewives can gain correct knowledge about edible oils, establish a healthy model and promote a healthy lifestyle. The status, baseline and knowledge and attitude questionnaires are designed based on KAP theory. During the intervention phase, we will provide different forms of effective intervention strategies at various stages of behavioural change based on social cognitive and SOC theories.

### K A P

This theory is one of the models used to change health-related behaviour. Knowledge (knowledge and learning) is the foundation, belief (belief and attitude) is the driving force and behaviour is the goal. Therefore, the target will produce corresponding beliefs and eventually change subjects’ behaviour after gaining health-related knowledge [30, 31]. Nutritional education is an important external condition for promoting the transformation of knowledge into behaviour.

### Social cognitive theory

Social cognitive theory is used to explain the social learning process [32, 33]. It focusses on cognitive factors, such as human beliefs, memories, expectations, motivations and self-enhancement. Social cognitive theory addresses the interactions among person, environment and behaviour. On the personal domain, the comic strip used in the intervention will provide participants with knowledge and skills regarding edible oils and cooking. Then, through observational learning, edible-oil consumption habits may improve during mealtime situations. Such improvement may increase self-efficacy and enhance behavioural capability with regard to cooking and edible-oil choices.

### SOC theory

The SOC theoretical model was proposed in 1982 by Prochaska and Di-Climente for their smoking cessation programme [34]. The theoretical basis of this model is that people’s behavioural change is a process, and each person who undergoes behavioural changes has varying needs and motivations.

This theory divides people’s behavioural changes into six stages: (1) precontemplation, (2) contemplation, (3) preparation, (4) action, (5) maintenance and (6) termination. The pace at which people move between the six stages varies, and different individuals should adopt varying cognitive and behavioural strategies to change their behaviour [35].

1. Precontemplation: during this stage, people may be unaware of the dangers of excessive edible-oil consumption
2. Contemplation: during this stage, people already intend to reduce their edible-oil intake, but have not yet started
3. Preparation: during this stage, people may have attempted to reduce their edible-oil intake and know about the benefits of reducing their edible-oil use, but do not know how to do it
4. Action: during this stage, the daily intake of edible oils can be controlled but only for less than 6 months
5. Maintenance: during this stage, the intake of edible oils has been successfully reduced for more than 6 months
6. Termination: during this stage, thorough control of edible-oil intake is achieved

### Study procedure

This study uses a combination of primary and secondary data. The quantitative cross-sectional studies will last for 4 weeks and will use WeChat as an intervention tool. A total of 800 housewives between the ages of 25 to 45 years will be recruited from different communities in Chongqing, China. A status survey will be conducted from September 2019 to November 2019. A previous study [36] has proven that age and education are associated with edible-oil intake behaviour. To eliminate the influence of confounding factors, 200 housewives with similar ages and educational levels will be selected to participate in a baseline survey. Subsequently, they will be divided randomly following a 1:1 ratio into an intervention group (group A) and a control group (group B) using computerised simple randomisation in SPSS. In the next 4 weeks, group A will receive health education through WeChat every 2 days, whereas group B will receive nothing. Each participant will then be given knowledge, attitude and behaviour tests immediately (immediate effect), 3 months (short-term effect) and 6 months (long-term effect) after the intervention. In accordance with the SOC model [35], the entire process of changing edible-oil intake behaviour among the housewives will be divided into six stages (Table 1). In addition, the changes in their behaviour stages and influencing factors will be analysed to evaluate the effects of the intervention on edible-oil intake. All subjects will read and sign a written informed consent form before their voluntary participation.

**Table 1:** Description of intervention methods corresponding to different stages

Stages	Intervention focus
Precontemplation	Spread the knowledge and benefits related to healthy oils
Contemplation	Encourage them to start trying to control oil intake
Preparation	Guide participants on how to use edible oils in a healthy manner
Action	Help participants identify factors that affect their persistence in decreasing oil intake
Maintenance	Self-motivation
Termination	\

### Randomisation and blinding

After the status survey, 200 housewives with similar ages and educational levels will be selected and divided randomly following a 1:1 ratio into an intervention group and a control group using SPSS. The study coordinator will be responsible for the randomisation and verification of the computer-generated treatment assignment with a randomisation table. All the investigators, except for the study coordinator, will be blinded. The participants will be instructed not to inform the other participants of their group assignment.

### Status survey

A self-designed survey questionnaire will be used to investigate the knowledge, attitude and behaviour related to edible oils of the 800 participants from Chongqing, China. Furthermore, the questionnaire can be used to screen participants who meet the inclusion criteria. The purpose of the questionnaire is to obtain the current status of and problems in edible-oil consumption among housewives in Chongqing. Questions related to edible-oil knowledge, attitude and behaviour are given 100 points. A high score corresponds to excellent knowledge and a positive attitude regarding edible-oil intake. Besides, in the questionnaire, we will ask who makes the decision on food choice in the household.

### Baseline survey

A self-designed questionnaire based on the KAP model will be used to acquire the general information from the participants. The questionnaire consists of a combination of demographic and knowledge items. The demographic items will ask for the personal information of the participants, including their age, educational level, occupation, family members and WeChat usage habit. The knowledge items are questions regarding edible-oil knowledge and intake behaviour. 7:55 a.m. (US) or 7.55 a.m. (UK

### Health education intervention

Group A will receive health education through WeChat at 8.00 p.m. every 2 days. A previous study [37] has proven that WeChat push frequency has an impact on information dissemination. When the WeChat platform is used to provide education regarding edible oils, attention should be given to the time and frequency of intervention to avoid inciting negative behaviour among the participants. Thus, we will provide intervention every 2 days. Previous studies [38] have confirmed that the rate of reading articles published on WeChat accounts peaks at 9.00 p.m., and thus, we will publish the content at 8.00 p.m. to allow subjects to better accept the intervention and help us improve its effectiveness. Moreover, we will collect weekly feedback from the participants using two self-designed questions through WeChat: ‘Are you interested in the contents this week?’ and ‘What do you suggest for next issue’s content?’ The feedback will help us assess the satisfaction of the participants, and consequently, improve the contents and methods of intervention.

In accordance with the Chinese Dietary Guidelines [10] and the Chinese Dietary Reference Intakes, health education can be provided through lively presentations with text, images, videos and audios. The WeChat public platform will be used as the communication tool of the intervention group for 4 weeks. Content will include information regarding the negative effects of excessive edible-oil intake, facts about edible oils, suggested reasonable amounts of edible-oil intake and lectures on healthy oil use guidelines provided by nutrition experts. Information about the differences between saturated and unsaturated edible oils (Table 2) and their composition and nutritional properties will be provided to aid in edible-oil selection according to individual needs. Questions for increasing public interaction will also be included, such as ‘Did you control your oil intake today?’ and ‘Do you pay attention to your edible-oil intake?’ An interactive platform will be set up to allow nutrition experts to answer participants’ questions in real time. The process will last for 4 weeks, and the intervention effects on edible-oil usage will be evaluated immediately, 3 months and 6 months after intervention.

**Table 2:** Fatty acid composition of common edible oils (%)

	Saturated fatty acid	Monounsaturated fatty acids (ω-9)	Polyunsaturated fatty acids		Trans fat
			Linoleic acid (ω-6)	Alpha-linolenic acid (ω-3)	
Olive oil	15	75	9	1	ND
Soybean oil	15	23	53	8	1
Canola oil	7	61	21	11	ND
Peanut oil	19	48	32	ND	0.6
Nutritional blend oil	9	28	60	ND	2
Lard	43	44	9	ND	ND

Note: ‘ND’ denotes no detection, and the content is below 0.05% [40]

### Knowledge, attitude and behaviour tests

The knowledge, attitude and behaviour tests will consist of two parts: a knowledge and attitude questionnaire survey and a behaviour test on edible-oil intake. The knowledge, attitude and behaviour tests will be administered to each participant during three face-to-face visits: immediately, 3 months and 6 months after intervention.

#### Knowledge and attitude questionnaire

Participants will answer a self-designed questionnaire, which includes knowledge regarding edible oils and intake attitude. The questionnaire is designed based on KAP, and it contains 30 items with only one correct answer. Each question is equivalent to 1 point. A high score corresponds to excellent knowledge and a positive attitude regarding edible-oil intake. Changes in participants’ knowledge and attitude will be assessed based on their final score.

#### Edible-oil intake behaviour test

Data on edible-oil intake will be obtained by conducting a continuous 3-day weighing survey on households, i.e. investigating the consumption of various edible oils within a family for 3 days. The survey time includes two working days and one rest day [39]. We will use the SF-400 kitchen scale (a calibrated bench-top scale; the maximum allowable amount is 5 kg and the sensitivity is 1 g) to measure the consumption of household edible oils [39]. The survey respondents are all household members and guests who consume cooking oil and condiments in the surveyed households. The daily consumption of individual edible oils is obtained using a family oil estimation algorithm. The decrease in the participants’ use of edible oils will be assessed according to the obtained data.

Daily consumption of oil intake estimates of each inhabitant (g/standard) = (3 days’ intake of edible oils by the surveyed households × standard daily number of people in a survey per household/standard number of people in all households)/3 [40].

### Quality control

To ensure the reliability of the survey data, the intervention programme will be established by the Department of Nutrition and Food Hygiene, School of Public Health and Management, Chongqing Medical University. The reliability and validity of the baseline and knowledge and attitude questionnaires will be tested.

A study manual will be provided before the survey, and training will be conducted among all the investigators. A standardised face-to-face questionnaire will be administered to the participants through the appropriate implementation and application of the unified method. Standardised experimental equipment and measurement methods will be used in the study, and kitchen scales will undergo quality control. To ensure that the weighing results of home cooking oil are accurate and reliable, the weighing range of the kitchen scales used in the survey will be set to 0–5 kg, with an accurate value of 1 g.

### Data management and entry

All the questionnaires will be collected, stored in a single file and regularly checked and rechecked. The questionnaires will be encoded and entered in a timely manner using the EpiData 3.1 double-entry test results.

### Analysis strategy

Statistical analyses will be conducted using IBM SPSS Statistics (version 22.0). Descriptive statistics will be calculated for all the variables under examination. Anthropometric measurements at the baseline will be analysed to examine the influence of the factors on edible-oil intake behaviour via multivariate regression analysis, with age, occupation and education level included in the model.

## Discussion

In accordance with the experiment design, the intervention group will receive 4 weeks of intervention to determine whether differences in edible-oil knowledge and intake behaviour exist between the intervention group and the control group. The experimental design will ensure balance among the confounding variables in each group and will eliminate the bias caused by the characteristics of the participants. Given that the enrolled participants should be good at using the WeChat app, the samples will exhibit this bias from the beginning. To avoid potential biases during the entire process, we will strictly follow the inclusion criteria when recruiting participants and use blind methods to collect data during the implementation phase. In addition, to increase the participation rate and reduce the selection bias, we will use children’s health as an entry point. Given that the participants are women between the ages of 25 to 45 years, most of them will have children in their family. In China, children are considered ‘emperors’ in the family. Most women care about their children’s health; thus, we will provide knowledge about edible-oil consumption among children during the intervention.

This study aims to encourage participants to decrease their use of edible oils and change their unhealthy behaviour. We hypothesise that group A will have higher awareness and willingness to decrease their use of edible oils than group B, which will indicate that the intervention based on social cognitive theory is effective. Moreover, the knowledge and attitude of participants will improve immediately after the intervention but will decrease considerably after 6 months. Such results would imply that the immediate effect of the intervention would provide the best results, whereas the long-term effect would be poor. Moreover, we believe that the intervention results may be affected by the duration of the intervention, as confirmed in previous studies [41]. A short intervention may show that the effect is inevident. In such a case, we can appropriately increase the duration of intervention in the follow-up study. Moreover, the traditional ideas of the participants are ingrained; even if they know that the excessive consumption of saturated edible oils are among the risk factors of chronic diseases, they will still not reduce their intake. A previous study [42] has confirmed that an increase in health knowledge is not always accompanied by changes in unhealthy behaviour and the transition from the intention stage to the action stage will take time. We speculate that this condition may also affect the outcome of our intervention. In addition, we speculate that the participants’ habits and economic status may also influence their edible-oil intake behaviour.

### Strengths and weaknesses

The present study exhibits strengths and weaknesses. This work will be the first intervention that will attempt to reduce the use of edible oils in China through WeChat. Moreover, the adoption of the three models, namely, KAP, social cognitive and SOC theories, will allow us to address the limitations of each model. The limiting factors of this study include the intervention that focusses on the family’s dietary decision-maker (i.e. housewives), without providing health education intervention to all family members. In addition, given the wide-spread use of WeChat, the participants may transmit the intervention to other people, through the app, without our permission. Therefore, we cannot guarantee that each participant in the control group will not receive knowledge regarding edible-oil intake from other sources during the intervention period.

In conclusion, this study should provide guidance on family health education and behaviour intervention regarding edible oils by increasing housewives’ knowledge regarding edible oils and encouraging them to use edible oils scientifically to promote overall health. This study can also be used as a reference for foreign studies. Through the evaluation of the intervention effects, changes in knowledge and behaviour regarding the edible-oil intake of the participants who received health education will validate our hypothesis and may encourage public officials to focus on health education and realise the value of the WeChat platform.

### Trial status

The recruitment phase of this study is scheduled from May 2019 to August 2019. Health education intervention will be conducted for 4 weeks for each participant. Follow-ups will be conducted over 6 months. Data analysis and evaluation will be performed after 12 months. The final results of this study will then be published.

## Additional files

Additional file 1: Standard Protocol Items: Recommendations for Interventional Trials (SPIRIT) 2013 Checklist: recommended items to address in a clinical trial protocol and related documents*. (DOC 125 kb)
